# Conduction system pacing for cardiac resynchronization therapy: a systematic review

**DOI:** 10.1093/europace/euag122

**Published:** 2026-05-19

**Authors:** Georgios Fotos, Nikolaos Ktenopoulos, Konstantinos Vlachos, Apostolos Christou, Maria Agelaki, Anastasios Apostolos, Athanasios Samaras, Justin Luermans, Kevin Vernooy, Konstantinos Tsioufis, Konstantinos Toutouzas, Dominik Linz, Serge Boveda

**Affiliations:** 1st Cardiology Department, Hellenic Red Cross Hospital, Athens, Greece; Cardiology Department, Bioclinic Athens, Athens, Greece; First Department of Cardiology, National and Kapodistrian University of Athens, Hippokration General Hospital of Athens, Athens 11527, Greece; Hôpital Cardiologique du Haut Léveque, CHU de Bordeaux, L'Institut de RYthmologie et Modelisation Cardiaque (LIRYC), Université de Bordeaux, Bordeaux, France; Electrophysiology Department, Hygeia Hospital, Erithrou Stavrou 4-5, Athens 15123, Greece; 1st Cardiology Department, Hellenic Red Cross Hospital, Athens, Greece; 1st Cardiology Department, Hellenic Red Cross Hospital, Athens, Greece; First Department of Cardiology, National and Kapodistrian University of Athens, Hippokration General Hospital of Athens, Athens 11527, Greece; Royal Brompton and Harefield Hospitals, Guy's and St Thomas’ NHS Foundation Trust, London, UK; 2nd Cardiology Department AUTh, Hippokrateion General Hospital of Thessaloniki, Thessaloniki, Greece; Department of Cardiology, Maastricht University Medical Centre and Cardiovascular Research Institute Maastricht, Maastricht, The Netherlands; Department of Cardiology, Maastricht University Medical Centre and Cardiovascular Research Institute Maastricht, Maastricht, The Netherlands; First Department of Cardiology, National and Kapodistrian University of Athens, Hippokration General Hospital of Athens, Athens 11527, Greece; First Department of Cardiology, National and Kapodistrian University of Athens, Hippokration General Hospital of Athens, Athens 11527, Greece; Department of Cardiology, Maastricht University Medical Centre and Cardiovascular Research Institute Maastricht, Maastricht, The Netherlands; Department of Biomedical Sciences, Faculty of Health and Medical Sciences, University of Copenhagen, Copenhagen, Denmark; Department of Heart Rhythm, Clinique Pasteur, Toulouse, France; Heart Rhythm Management Centre, European Reference Networks Guard-Heart, Universitair Ziekenhuis Brussel, Heart Rhythm Research Brussels, Postgraduate Program in Cardiac Electrophysiology and Pacing, Vrije Universiteit Brussel, Brussels, Belgium

**Keywords:** His bundle pacing, Left bundle branch area pacing, Heart failure, Left bundle branch block, Biventricular pacing, Conduction system pacing

## Abstract

Biventricular pacing (BVP) remains the standard method for delivering cardiac resynchronization therapy (CRT) in patients with heart failure and left bundle branch block (LBBB). Despite its established role, BVP is a non-physiological pacing approach and is limited by factors such as anatomical constraints of the coronary sinus and a substantial proportion of non-responders. Conduction system pacing (CSP), including His bundle pacing and left bundle branch area pacing, has emerged as a more physiological alternative aimed at optimizing CRT outcomes. Early studies suggest that CSP may achieve superior electro-mechanical ventricular synchrony compared with BVP. Nevertheless, evidence from large-scale randomized controlled trials remains scarce, and challenges related to procedural complexity and insufficient long-term outcome data currently prevent CSP from replacing BVP as the first-line CRT strategy. This systematic review synthesizes the existing literature on CSP in CRT, explores its potential applications across different clinical contexts, and discusses the ongoing controversies and future prospects of this evolving technique.

## Graphical Abstract

**Graphical Abstract:**
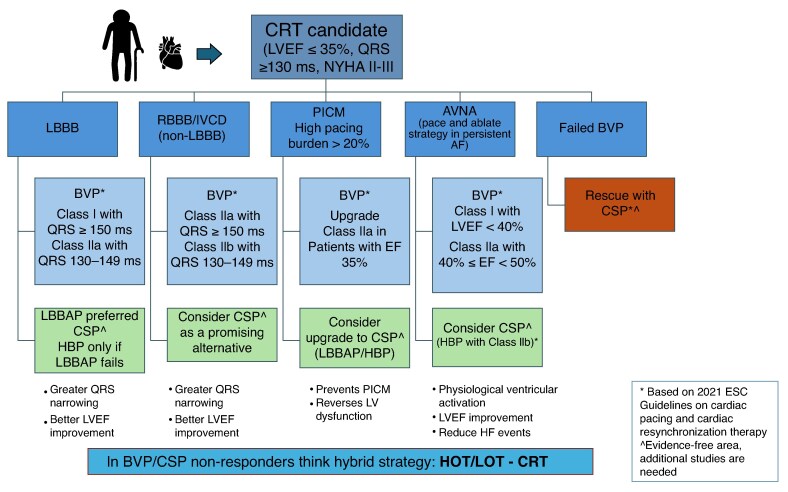
Proposed algorithm for conduction system pacing (CSP) in cardiac resynchronization therapy (CRT). In HF with wide QRS (>130 ms), biventricular CRT (BiV-CRT) is standard for LBBB; left bundle branch area pacing is the preferred CSP, with His bundle pacing an option in case of LBBAP failure. CSP may also be used in right bundle branch block (RBBB), intraventricular conduction delay, pacing-induced cardiomyopathy (PICM), and atrial fibrillation (AF) with atrioventricular nodal ablation (AVNA), where it is an alternative to standard BiV-CRT. Non-responders to BiV-CRT or CSP may benefit from His-optimized CRT (HOT-CRT) or left bundle branch area pacing–optimized CRT (LOT-CRT). When the CS lead implantation fails, it is proposed to perform CSP (LBBAP over HBP).

## Introduction

Cardiac resynchronization therapy (CRT) via biventricular pacing (BVP) remains a cornerstone completing guideline-directed medical therapy for heart failure (HF) with reduced ejection fraction (HFrEF), particularly in the presence of left bundle branch block (LBBB).^1^ Although BVP improves symptoms, survival, and ventricular remodelling; it represents a non-physiological pacing strategy, merging depolarization wavefronts from the left ventricular (LV) epicardium and the right ventricular (RV) endocardium, thus yielding partial resynchronization with modest QRS narrowing.^2^ Procedural limitations, including challenging coronary sinus (CS) anatomy, elevated capture thresholds, and phrenic nerve stimulation, may reduce therapeutic effectiveness, while up to one-third of patients fail to respond.^3^

**Figure 1:**
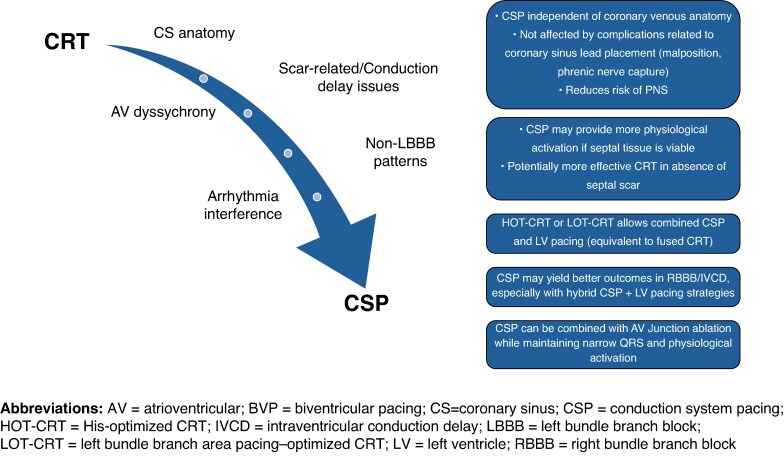
Challenges associated with biventricular pacing and potential solutions offered by conduction system pacing (CSP).

In this context, conduction system pacing (CSP), encompassing His bundle pacing (HBP) and left bundle branch area pacing (LBBAP), has emerged as a promising physiological alternative, engaging the native His–Purkinje system to restore near-normal electromechanical activation (*Figure 1*).^4,5^ While guidelines regarded CSP as a bail-out option, the 2025 ESC/EHRA consensus reframes it as a physiologic pacing modality, generally favouring LBBAP over HBP.^6^ This systematic review synthesizes available literature on CSP in CRT, outlining clinical applications, unresolved challenges, and potential future directions.

## Physiological and haemodynamic benefits of conduction system pacing

**Figure 2:**
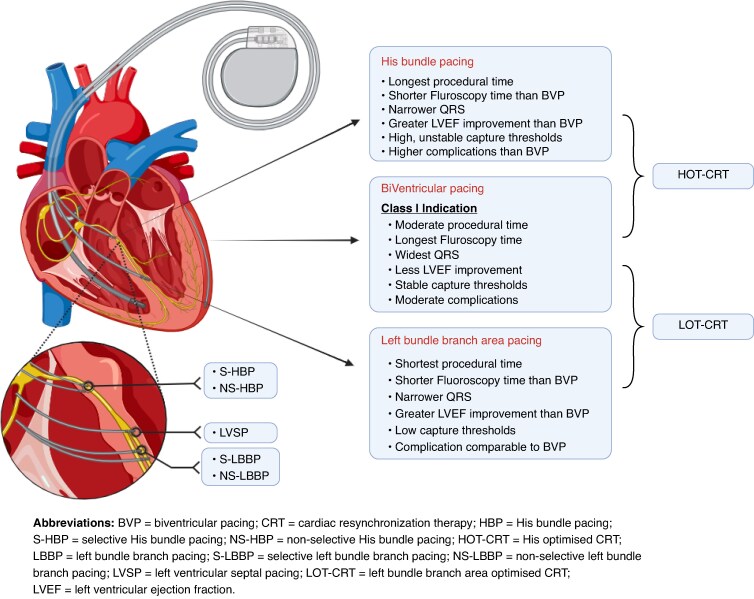
Types of CSP, procedural and follow-up outcomes with different CRT techniques based on available evidence.

Electrophysiologically, HBP can be classified into selective (S-HBP), capturing only the His bundle, and non-selective (NS-HBP), capturing also the adjacent septal myocardium. Although S-HBP offers the most authentic form of physiological resynchronization, it faces significant hurdles such as elevated capture thresholds, low signal amplitude, ventricular undersensing, and *P*-wave oversensing.^7^ LBBAP, similarly, can be classified into selective left bundle branch pacing (S-LBBP), non-selective left bundle branch pacing (NS-LBBP), and left ventricular septal pacing (LVSP), the latter involving myocardial capture of the LV aspect of the IVS without direct conduction system activation.^8^ LBBAP, first described by Huang *et al.*, generally provides lower capture thresholds, higher signal amplitudes, reduced risk of oversensing atrial electrograms, and a broader target area than HBP, although delayed RV activation remains a potential limitation^9,10^ (*Figure 2*).

The ventricle’s activation, first elucidated in the late 1960s, begins in the endocardium along the left aspect of the interventricular septum, at the level of the left bundle branch, and then propagates apicobasal via Purkinje network terminations embedded within the endocardium.^11^ CRT aims to correct the delayed activation of the LV free wall, most often correlated with LBBB, narrowing the QRS complex, which is linked to improved outcomes.^12^ Acute haemodynamic assessments indicate that CSP may yield superior electromechanical resynchronization than BVP. Specifically, HBP produced greater QRS narrowing, shorter LV activation times, reduced dyssychrony index, and enhanced arterial pressure compared to BVP, and provided better ventricular resynchronization than LBBAP due to delayed RV activation with the latter.^13^ Preliminary data also suggest that normalization of ventricular activation with CSP may improve diastolic function.^14^

Beyond resynchronization, emerging observational data suggest that CSP strategies (especially LBBAP) may also be associated with a lower incidence of ventricular arrhythmic events compared with BVP, although the clinical relevance of this association remains to be established.^15^

Multiple mechanisms have been proposed to explain how CSP improves ventricular and electrical synchrony and activation in patients with bundle branch block (BBB), with QRS narrowing being a supportive marker. These benefits derive from correction of proximal conduction block within the His bundle fibres.^16^ In support of this, Upadhyay *et al.*, after mapping the LV septum in LBBB patients, identified a proximal conduction block in two-thirds, which can be overcome by pacing distally to the obstruction. Conversely, in intraventricular conduction delay (IVCD), where conduction disease extends distally, HBP was less effective.^17^ Other potential mechanisms for BBB correction via CSP include the virtual electrode effect (whereby pacing at higher output proximal to the site of the block may capture distal conduction fibres), inter-bundle transverse connections, and retrograde activation of the HB and right bundle branch (RBB), particularly during LBBAP.^18,19^

HBP achieves the synchronous biventricular activation, preserving native conduction, whereas LBBAP provides near-normal LV activation with an incomplete RBBB pattern. Both offer superior mechanical synchrony compared to BVP, though further evidence is required to elucidate the electromechanical consequences of CSP and their impact on CRT outcomes.^20^

By engaging the His–Purkinje system, CSP, particularly LBBAP, supports a more physiological pattern of ventricular activation than BVP, reflected by greater electrical synchrony and QRS narrowing. These mechanistic advantages have been associated with favourable electrical and remodelling outcomes in early clinical studies. Nevertheless, the available evidence is largely derived from observational and small randomized studies with limited follow-up and heterogeneity in patient selection and implantation techniques, while long-term data on lead performance and safety remain limited. Consequently, despite its increasing adoption, particularly in cases of CS lead failure or suboptimal response to BVP, CSP should currently be regarded as a complementary rather than a substitutive strategy within contemporary CRT.^21^

## Methods

This systematic review was conducted in accordance with the Preferred Reporting Items for Systematic Reviews and Meta-Analyses (PRISMA) 2020 statement.^22^  *A priori* methods were defined before study selection. The study protocol was registered prospectively in the International Prospective Register of Systematic Reviews (PROSPERO) (CRD420251176588).

### Eligibility criteria

We included studies enrolling adults (≥18 years) with HF and an indication for CRT, including *de novo* CRT candidates and patients undergoing CSP as rescue or upgrade after failed/non-response to biventricular CRT (BiV-CRT). Eligible interventions were CSP strategies used for CRT, categorized as: (i) HBP-based CRT, including HBP-CRT and His-optimized CRT (HOT-CRT), and (ii) LBBAP-based CRT, including LBBAP-CRT and left bundle branch optimized CRT (LOT-CRT). Comparators included conventional BiV-CRT. We included randomized controlled trials (RCTs) and prospective or retrospective observational cohorts. None of the included RCTs were fully double-blinded, given the nature of CRT procedure. Case reports, editorials, expert opinions, animal studies, paediatric studies, and abstracts without sufficient outcome data were excluded. No restriction was placed on follow-up duration or publication language. The full PICO criteria are presented in the Supplementary File.

### Information sources and search strategy

A comprehensive literature search was performed in PubMed/MEDLINE, the Cochrane Central Register of Controlled Trials (CENTRAL), and Scopus from database inception to 15 November 2025. The full search algorithms for each database are provided in the Supplementary File. Reference lists of included studies and relevant reviews were manually screened to identify additional eligible studies.

### Study selection

**Figure 3:**
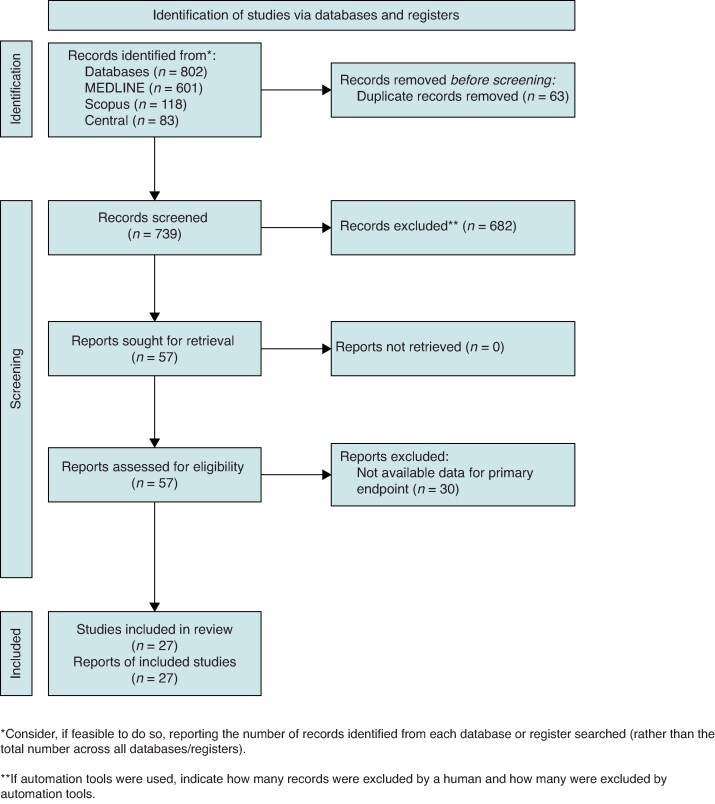
PRISMA 2020 flow diagram for new systematic reviews, which included searches of databases and registers only.

All records were imported into the Rayyan software for de-duplication and screening, and duplicates were removed.^23^ Two reviewers (G.F. and N.K.) independently screened titles and abstracts for potential eligibility. Full texts of potentially relevant articles were then assessed independently by the same reviewers against inclusion criteria. Disagreements were resolved through discussion and, when necessary, by adjudication with a third reviewer (K.T.). Reasons for exclusion at the full-text stage were recorded. The study selection process is summarized in the PRISMA flow diagram (*Figure 3*).

### Synthesis of results

Given the heterogeneity in study designs, patient populations, CSP implantation techniques, and reported endpoints, quantitative pooling was not performed. We undertook a structured narrative synthesis guided by SWiM principles.^24^ Studies were grouped *a priori* by CSP category: (i) HBP/HOT-CRT and (ii) LBBAP/LOT-CRT and further stratified where data allowed by clinical setting and baseline conduction pattern (LBBB vs. non-LBBB). For each outcome, we summarized the direction and magnitude of effects across studies, prioritizing randomized and prospective data when available. Consistency of findings, plausible mechanistic explanations, and the influence of risk of bias were considered in drawing conclusions.

## Cardiac resynchronization therapy in left bundle branch block

### His bundle pacing for cardiac resynchronization therapy in left bundle branch block

Current guidelines recommend BVP with a Class I indication for patients with symptomatic HF, QRS ≥150 ms with LBBB morphology, and a left ventricular ejection fraction (LVEF) ≤35%, despite optimal medical therapy.^1^ RCTs support BVP’s ability to improve survival, reduce HF hospitalizations, and provide durable clinical benefit. Nevertheless, its non-physiological ventricular activation pattern, variability in patient response, and the need for a three-lead system have spurred interest in HBP as a physiological CRT alternative. Growing data suggest that when successful, HBP-CRT is feasible and associated with improved LVEF and New York Heart Association (NYHA) functional status.^25^

Barba-Pichardo *et al.* in 2013 first described HBP-CRT in patients with advanced HF in whom CS lead placement was not possible. Among 16 such patients, conduction abnormalities were corrected in 13 (81%), and nine of these (69%) achieved permanent resynchronization with HBP, accompanied by improvements in functional class and echocardiographic measures of LV function.^26^

In 2015, Lustgarten *et al.* conducted a crossover trial comparing HBP and BVP in 29 CRT candidates. Resynchronization was achieved in 21 patients (72%), who were then randomized in a single-blinded manner to receive either HBP or BVP for 6 months, followed by crossover to the alternate modality for another 6 months. Of the 12 patients completing both phases, both pacing approaches led to significant gains in LVEF, NYHA class, and 6-min walk distance.^27^

A larger, multicentre retrospective study by Sharma *et al.* evaluated 106 patients undergoing HBP-CRT, 73 as a primary strategy and 33 as rescue therapy, with 90% implant success rate. Over a 14-month follow-up, both groups demonstrated significant QRS narrowing (from 157 ± 33 ms to 117 ± 18 ms), improved LVEF, and enhanced NYHA class.^28^

To date, only two small pilot RCTs have directly compared HBP and BVP. The HIS-SYNC trial randomized 41 patients (21 to HBP-CRT, 20 to BVP-CRT) and followed them for 6 months. Crossovers occurred in both directions −48% from HBP to BVP due to inadequate QRS narrowing, and 25% from BVP to HBP due to failed coronary venous lead placement, highlighting the complementary nature of the techniques. The trial suggested a trend towards greater QRS shortening with HBP, though LVEF improvements were similar, with a median of 6.2 months.^29^

The His-Alternative trial randomized 51 HF patients with LBBB to HBP-CRT or BVP-CRT. His-corrective pacing was achieved in 72% of HBP patients. At 6 months, LVEF improvement was comparable between groups, though pacing thresholds were significantly higher with HBP, particularly during follow-up. High crossover rates, largely due to inadequate QRS narrowing, were noted in the HBP arm.^30^ In a substudy of the His-Alternative, both BVP-CRT and HBP-CRT markedly reduced mechanical dyssynchrony without a major difference between the two groups. Additionally, global longitudinal strain improved comparably in the two groups, with no differences in regional strain. These findings support the concept that HBP-CRT achieves at least equivalent mechanical resynchronization and contractile improvement compared with BVP-CRT.^31^

The studies discussed above, along with all eligible investigations on HBP included in this systematic review, are summarized in *Table 1*.^27,28,30–38^

**Table 1:** Summary of included studies evaluating His bundle pacing–based CRT (HBP-CRT) and His-optimized CRT (HOT-CRT)

Author/year	Number of patients	Study type	Group/CRT type	Key findings	Safety/complications
Lustgarten D.L. *et al.*, 2015^27^	29	RCT	HBP vs. BVP	Comparable haemodynamic and clinical results	No major events
Sharma P.S. *et al.*, 2018^28^	106	Observational comparative	HBP vs. BVP	HBP feasible, improved synchrony	Low complication rate
Upadhyay G.A. *et al.*, 2019	41	RCT	HBP vs. BVP	Comparable response with CRT; better electrical synchrony	Slightly higher pacing threshold
Vinther *et al.*, 2021^30^	50	RCT	HBP vs. BVP	Successful impantation: HBP 72% vs. CRT 96%; improved electrical synchrony	No major events
Moriña-Vázquez P. *et al.*, 2022^33^	103	Observational prospective (with historical series of CRT)	HBP vs. BVP	HBP: better LVEF improvement, similar thresholds	No major events
Huang W. *et al.*, 2022^34^	120	Observational comparative	HBP vs. BVP	Better CRT response and EF improvement	Few complications
Højgaard E. V. *et al.*, 2023^31^	45	RCT substudy	HBP vs. BVP	Improved longitudinal contractile function	No major events
Vijayaraman P. *et al.*, 2019^35^	27	Observational retrospective	HOT-CRT vs. BVP	HOT-CRT: narrower paced QRS, better LVEF and NYHA status	Comparable
Deshmukh *et al.*, 2020^36^	21	Observational retrospective	HOT-CRT vs. BVP	Paced QRS: HOT-CRT 110 ± 14 ms vs. BiV: 141 ± 15 ms	No major events
Zweerink A. *et al.*, 2021^37^	19	Observational prospective	HOT-CRT vs. BVP	HOT-CRT improved LVAT	No major events
Vijayaraman P. *et al.*, 2023^38^	100	Prospective RCT	HOT-CRT vs. BVP	ΔLVEF: HOT-CRT 12.4 ± 7.3% vs. BiV 8 ± 10.1%	Fewer complications with HOT-CRT

*Abbreviations*: BVP, biventricular pacing; CRT, cardiac resynchronization therapy; EF, ejection fraction; HBP, His-bundle pacing; HOT-CRT, His-optimized CRT; LVEF, left ventricular ejection fraction; NYHA, New York Heart association; RCT, randomized clinical trial

Current guidelines do not yet endorse HBP as a primary CRT strategy, but recommend its use (Class IIb) when CS lead implantation fails.^1^ Although promising as a physiological CRT modality, larger trials are necessary to validate its long-term benefits and potentially expand its role in clinical practice. Persistent limitations, such as high capture thresholds, low R-wave amplitudes, atrial oversensing, inability to correct distal conduction block, along with long term lead issues requiring revision in 7–11% of cases, continue to constrain its use.^8^ Consistent with this, the 2025 ESC/EHRA consensus acknowledges HBP as a viable option for CRT in LBBB when proximal block correction is achievable, but generally favours LBBAP as the more practical CSP approach due to superior implant success and long-term stability^6^ (*Figure 2*).

### Left bundle branch area pacing for cardiac resynchronization therapy in left bundle branch block

Procedural limitations of HBP have driven interest in LBBAP as an alternative approach.^39^ The concept of deep septal pacing, first demonstrated by Mafi-Rad *et al.*^40^ and clinically applied by Huang *et al.* in 2017 in a patient in whom HBP failed to correct LBBB, even at maximal output. By advancing the lead distally from the His bundle towards the ventricular septum, they successfully captured the proximal LBB trunk at a lower, more stable threshold, achieving optimal QRS narrowing and a marked improvement in LVEF at 1-year follow-up.^9^

Subsequent observational studies have reported high implantation success rates (80–97%), clinical and echocardiographic improvement, with particularly high expectations for its role in CRT among patients with HF and LBBB.^41–45^ Furthermore, in an international multicentre registry, LBBAP was shown to be an effective rescue option for patients with CS lead failure or non-response to BVP, reducing QRS duration from 170 ± 28 to 139 ± 25 ms and improving LVEF from 29 ± 10% to 40 ± 12%.^46^

Randomized data comparing LBBAP and BVP remain limited. In a small trial of 40 patients with non-ischaemic cardiomyopathy (NICM) and LBBB, LBBAP was associated with a significantly greater LVEF improvement at 6 months (mean difference: 5.6%; 95% CI: 0.3–10.9), with larger reductions in LV end-systolic volume and NT-proBNP, with no significant differences in functional capacity, QRS duration change, or response rates.^47^ Similarly, in 21 HF patients (predominantly NICM), LBBAP achieved a greater reduction in QRS duration (−11 ms; 95% CI: −17 to −4), QRS area (−85 μVs; 95% CI: −113 to −56), and a higher dP/dtmax (6%; 95% CI: 2–9%) compared to BVP.^48^

Pooled analyses suggest potential prognostic advantages. In a large multicentre cohort of patients with HFrEF undergoing CRT, CSP, via either HBP or LBBAP, was associated with a lower risk of all-cause mortality or HF hospitalization compared with BVP, predominantly in LBBB patients.^49^ Network meta-analyses indicate that both CSP techniques achieve greater QRS narrowing and LVEF improvement than BVP, with LBBAP offering the additional advantages of significantly lower pacing thresholds, higher R-wave amplitudes, minimal far-field atrial oversensing, and a broader target area for a faster procedural learning curve.^50^ In a recent non-inferiority RCT of 70 patients, LBBAP demonstrated greater implant success rates (82% vs. 57% for HBP) while achieving comparable reductions in LV activation time (LVAT), reverse remodelling, and 6-month mortality or HF hospitalization.^51^

The large I-CLAS observational study compared 1778 HF patients (LVEF ≤35%) treated with either BVP or LBBAP. LBBAP achieved shorter QRS duration and significantly greater LVEF improvement, and propensity-matched analysis found lower rates of the composite endpoint of HF hospitalization and all-cause mortality in the LBBAP group.^52^ Finally, the CSP-SYNC study directly compared LBBAP with BiV pacing. At 6 months, LBAAP was non-inferior to BiV pacing for LVEF improvement, with a mean absolute increase of 5.6% favouring LBBAP (95% CI 1.6–9.5). Both groups achieved comparable reductions in LV end-systolic volume, improvements in functional capacity and NYHA class, supporting LBBAP as an effective alternative to conventional CRT in patients with typical LBBB.^53^

These findings were further supported by the CONSYST-CSP trial, which provided the largest randomized comparison between CSP and BVP, enrolling 134 patients with 12-month follow-up. CSP demonstrated non-inferiority to BVP for the composite endpoint of death, HF hospitalization, or lack of echocardiographic response, but secondary outcomes including QRS narrowing, NYHA improvement, and LV remodelling were comparable between the two groups.^54^

The PhysioSync-HF is one of the largest multicentre RCTs comparing CSP with BVP-CRT. A total of 179 patients with HFrEF (LVEF ≤35%), primarily due to dilated cardiomyopathy, LBBB ≥130 ms, and NYHA Class II–III, were randomized to CSP or BVP-CRT. At 12-month follow-up, CSP did not complete the criteria of non-inferiority to BVP for the hierarchical composite primary endpoint of all-cause mortality, HF hospitalization, urgent HF visit, and LVEF change.^55^

Conversely, the HeartSync-LBBP trial, the largest to date multicentre RCT, provides robust evidence in favour of LBBAP in a specific HF phenotype. Enrolling 200 patients mainly with NICM, reduced EF ≤35%, LBBB, and NYHA Class II–III, this trial demonstrated that LBBAP yielded superior outcomes compared with BVP for the primary composite endpoint. Although there was no statistically significant difference in all-cause mortality or echocardiographic findings, the LBBAP group exhibited a significantly lower risk of hospitalizations due to HF, a lower capture threshold, and better functional capacity.^56^

The available data on LBBAP described in this section, along with all the eligible studies identified in this systematic review, are summarized in *Table 2*.^47,48,51,53–67^

**Table 2:** Summary of included studies evaluating left bundle branch area pacing–based CRT (LBBAP-CRT) and left bundle branch optimized CRT (LOT-CRT)

Author/year	Number of patients	Study type	Group/CRT type	Key findings	Safety/complications
Liang Y. *et al.*, 2022^48^	21	Multicentre/crossover	LBBAP vs. BVP	Greater reduction in paced QRS, haemodynamic improvement	No major events
Pujol-Lopez M. *et al.*, 2022^51^	70	**RCT**	CSP (mainly LBBAP) vs. BVP	Change in LVAT at 6 months: CSP similar to BVP	No major events
Chen X. *et al.*, 2022^66^	85	Observational prospective	LBBAP vs. BVP	Greater QRS reduction and LVEF improvement; reduced composite endpoint (HF/death)	No major events
Wang *et al.*, 2022^47^	40	**RCT**	LBBAP vs. BVP	ΔLVEF at 6 months: 5.6% (significant)	No major events
Vijayaraman P. *et al.*, 2023^38^	1778	Observational retrospective	LBBAP vs. BVP	26% reduction in composite death/HF admissions	No major events
Diaz J. C. *et al.*, 2023^58^	371	Observational prospective	LBBAP vs. BVP	38% reduction in composite endpoint	No major events
Diaz J. C. *et al.*, 2024^57^	415	Observational prospective	LBBAP vs. LVSP vs. BVP	LBAAP reduced significantly composite outcomes; LVSP comparable to BVP	No major events
Palmisano P. *et al.*, 2024^60^	668	Observational comparative	LBBAP vs. BVP	LBBAP associated with similar CRT efficacy, lower complications vs. BVP	Device-related complications lower in LBBAP (4.3% vs. 12.9%); BVP independently associated with higher complication risk
Zhu H. *et al.*, 2024^61^	259	Observational prospective	LBBAP vs. BVP	LBBAP achieved better clinical outcomes (death/HF hospitalizations)	No major events
Schroff P.J. *et al.*, 2024	101	Observational comparative	LBBAP vs. BVP	Similar with CRT response but with significant lower pacing thresholds; better electrical synchrony	No major events
Zizek *et al.*, 2025	270	**RCT**	LBBAP vs. BVP	LBBAP reduced HF admissions; improved LVEF and QRS width	No major events
Pujol-Lopez *et al.*, 2025^51^	134	**RCT**	CSP (mainly LBBAP) vs. BVP	Non-inferior to BVP for composite death/HF/ΔLVEF; QRS shortening, NYHA improvement	No major events
Morcos R. *et al.*, 2025^63^	2579	Observational prospective	LBBAP vs. BVP	LBBAP: shorter paced QRS; lower risk of the composite endpoint (death/HF); lower HF admissions	Lower complications in LBBAP arm, lower arrhythmic risk
Zimerman A. *et al.*, 2026^55^	173	**RCT**	LBBAP vs. BVP	Inferior to BVP for composite death/HFH/ΔLVEF; comparable improvement in QRS duration and functional capacity	More complications in LBBAP arm (10 vs. 7); 3 deaths in the same arm
Chen X. *et al.*, 2026^56^	200	**RCT**	LBBAP vs. BVP	LBBAP superior to BVP for composite death/HFH; similar echocardiographic response; better functional capacity in LBBAP arm	No major complications in LBBAP arm; one lead dislodgement and one increase in pacing threshold in BVP arm
Jastrzebski *et al.*, 2022^67^	91	Observational prospective	LOT-CRT vs. BVP	Narrower QRS vs. BVP and LBBAP, improved LVEF > 3 months, improved NYHA	No major events
Feng *et al.*, 2022^64^	21	Observational prospective	LOT-CRT vs. BVP	Narrower QRS, ΔLVEF > 9 months	No major events
Jastrzebski *et al.*, 2024^65^	48	Crossover observational	LOT-CRT vs. LBBAP vs. BVP	Advanced conduction diseases: LOT-CRT and BVP > LBBAP haemodynamic benefit	No major events

*Abbreviations*: BVP, biventricular pacing; CRT, cardiac resynchronization therapy; CSP, conduction system pacing; HF, heart failure; HFH, heart failure hospitalization; LVEF, left ventricular ejection fraction; LBBAP, left bundle branch area pacing; LOT-CRT, left bundle branch-optimised CRT; LVAT, left ventricular activation time; LVSP, left ventricular septal pacing; NYHA, New York Heart association; RCT, randomised clinical trial.

At present, international guidelines do not recommend LBBAP for routine CRT, but early data suggest electromechanical and clinical improvements. In line with this, the 2025 ESC/EHRA consensus statement highlights LBBAP as the more practical and generally preferred CSP modality for CRT in LBBB, especially when CS cannulation fails, owing to its higher implant success and stable long-term performance.^6^ The divergent findings between major trials such as PhysioSync-HF and HeartSync-LBBP highlight the existing heterogeneity across randomized comparisons and underscore the need for larger, long-term RCTs focusing on clinical endpoints to determine whether LBBAP should be adopted as a preferred alternative to BVP in suitable CRT candidates (*Figure 2*).

## CSP-CRT beyond left bundle branch block

Several conduction disorders beyond LBBB can result in ventricular dyssynchrony and may be amenable to CRT via physiological pacing strategies.

### Right bundle branch block and intraventricular conduction delay

Traditional BVP-CRT has failed to demonstrate a clinical benefit in RBBB patients, as LV stimulation via the CS does not directly address delayed RV activation. Conversely, both HBP and LBBAP may overcome this, improving QRS duration, LVEF, and New York Heart Association (NYHA) class.^28,68^ For patients with IVCD, where conduction system abnormalities coexist with intramyocardial conduction delays, optimal resynchronization may be achieved by combining CSP with LV epicardial pacing via BVP [His-optimized CRT (HOT-CRT) and LBBAP-optimized CRT (LOT-CRT)], improving symptoms, electrocardiographic parameters, and echocardiographic indices.^67^

The HOPE-HF trial evaluated AV-optimized HBP vs. no pacing in HF patients with LVEF ≤40%, a prolonged PR, and either a QRS ≤140 ms or RBBB. While peak oxygen uptake did not improve, quality of life scores increased significantly, suggesting a role for HBP in HF patients with first-degree AV block.^69^ Although no RCTs have been conducted in non-LBBB patients, the 2025 ESC/EHRA consensus highlights that in these patients, CSP may be appropriate in selected CRT candidates.^6^

### Treatment and prevention of pacing-induced cardiomyopathy

Conventional right ventricular pacing (RVP) produces non-physiological ventricular activation, predisposing to pacing-induced cardiomyopathy (PICM) with progressive systolic and diastolic dysfunction. Upgrading patients from RVP to CSP (HBP or LBBAP) can significantly narrow QRS duration and improve LVEF, partially reversing electrical and structural changes caused by long-term RVP.^70^ Furthermore, in pacemaker recipients with a high anticipated ventricular pacing burden (>20%), initial CSP implantation prevents PICM, estimated risk of around 12%, and reduces the composite risk of death, HF hospitalization, or subsequent upgrade to BVP.^71^ The 2025 ESC/EHRA consensus states that the use of CSP (HBP or LBBAP) may be appropriate to prevent or reverse PICM, emphasizing technique selection based on anatomical and conduction considerations.^6^

### Pace and ablate

Atrioventricular node ablation (AVNA) in patients with atrial fibrillation (AF) creates pacing dependency, thereby optimizing rate control and enhancing CRT response.^72^ In this setting, continuous RVP is associated with an increased risk of PICM, particularly in patients with pre-existing LV dysfunction.^72^ CSP offers a potential advantage by maintaining physiological ventricular activation in AVNA recipients at risk for PICM, with observational studies confirming its feasibility (higher success rates with LBBAP), improved QRS duration, LVEF, and reduced mortality and HF hospitalizations, when compared with RVP or BVP,^73^ In a recent multicentre, prospective, randomized crossover trial of 50 patients undergoing AVNA yielded, a modest but significant improvement in LVEF in patients with persistent AF, LVEF ≤40%, and narrow QRS was observed, compared to BVP.^34,74^ According to the 2025 ESC/EHRA consensus, the use of CSP may be appropriate in selected AVNA recipients as a strategy to maintain physiological ventricular activation and reduce the PICM risk, with technique selection guided by anatomical and procedural considerations.^6^

## Combination of conduction system pacing with conventional cardiac resynchronization therapy

In advanced cardiomyopathy, concomitant LBBB and IVCD may occur together, exacerbating LV dyssynchrony, as LBBB forces LV activation to depend on prolonged myocardial conduction pathways, while IVCD adds further delay to myocardial activation. In addition, one-third of BiV-CRT patients do not respond to CRT therapy, which is reflected by QRS widening rather than narrowing and is associated with poor prognosis.^12^ In those patients, CSP can improve LV activation with a narrower QRS by combining pacing of the specialized conduction system with sequential LV epicardial pacing.^75^

Several observational studies have evaluated His-optimized CRT (HOT-CRT). In a multicentre, retrospective series of 27 patients with LBBB/IVCD and HF (NYHA III-IV, LVEF ≤35%), HBP alone produced only partial or minimal QRS narrowing compared with baseline. HOT-CRT provided greater electrical resynchronization than either conventional BVP or HBP alone and a greater QRS shortening than BVP (34% vs. 11%), along with echocardiographic and clinical improvements.^37^

The HOT-CRT randomized controlled trial enrolled 100 patients to either HOT-CRT or BVP. HOT-CRT demonstrated higher implantation success (96% vs. 82%), greater QRS narrowing (164 ± 26 ms to 137 ± 20 ms vs. 166 ± 28 ms to 141 ± 19 ms), and LVEF increase at 6 months (12.4 ± 3.0% vs. 8.0 ± 10.1%) and less frequent complications (6% vs. 20%). Crossover to HOT-CRT occurred in 18% of patients following unsuccessful CS lead implantation, primarily due to anatomical or procedural limitations. Fluoroscopy time and total procedure duration were similar between groups.^38^

LBB area pacing–optimized CRT (LOT-CRT) has similarly shown benefit. In a multicentre observational study (*n* = 112), the procedural success rate was 81%. LOT-CRT achieved superior QRS narrowing compared with BVP or LBBP alone (21% vs. 7% vs. 11%). Significant gains were also seen in LVEF (28 ± 10% to 37 ± 12%) and NYHA class (2.9 ± 0.6 to 1.9 ± 0.6).^67^

Additionally, the CSPOT trial (NCT04905290) compares BVP, LBBP, and LOT-CRT at the time of implantation. Primary outcomes such as electrical resynchronization and haemodynamic change measured by LV dP/dtmax have been reported. LOT-CRT produced the greatest QRS shortening and a similar increase in LV dP/dtmax compared with BVP, while surpassing LBBP alone, especially in patients with QRS ≥171 ms and in cases where only deep septal pacing was used. In patients with IVCD, QRS narrowing was less pronounced than in LBBB, though LV dP/dtmax improvements were comparable.^65^ Six-month secondary endpoints on LVEF, LV remodelling, and clinical endpoints are pending publication.

Building on hybrid resynchronization strategies such as HOT/LOT-CRT, emerging approaches including bifascicular pacing have been proposed as multipoint left CSP techniques aiming to further optimize ventricular activation, although current evidence remains preliminary.^76^

The current evidence on HOT-CRT and LOT-CRT, identified through this systematic review, is comprehensively summarized in *Tables 1* and *2*, respectively.

## Ongoing clinical trials

The left vs. left randomized trial (NCT05650658) is the largest ongoing head-to-head comparison of CSP and BVP in CRT candidates, enrolling 2136 participants with a minimum follow-up of 3 years, to assess superiority for the primary composite endpoint of death and HF hospitalization, with completion expected in 2029.

The His-Bundle Corrective Pacing in Heart Failure trial (NCT05265520) is evaluating HBP-enhanced CRT vs. conventional BVP-CRT in HF patients with RBBB to determine clinical efficacy and mechanisms of benefit.^75^

Until more robust randomized evidence on hard clinical outcomes is available, and given that the most reported benefits of CSP relate to surrogate endpoints, CSP, particularly LBBAP, should be considered a valuable alternative or rescue option strategy, when conventional CRT is not feasible or fails to deliver adequate resynchronization, provided that left bundle capture can be achieved with meaningful QRS narrowing, in line with the 2025 ESC/EHRA consensus.^6^

## Safety and complications of conduction system pacing

Despite its physiological rationale, the broader implementation of CSP in CRT has raised concerns related to procedural complexity, lead performance, and long-term safety. Evidence addressing complications is largely derived from multicentre observational cohorts, registry-based analyses, and meta-analyses, as RCTs have not been specifically designed to assess safety endpoints.

HBP is limited by the anatomical characteristics of the His Bundle, which is located in a narrow and often fibrotic region. Consequently, HBP is frequently associated with higher acute and chronic capture thresholds, low R-wave amplitudes, and a higher susceptibility to atrial oversensing and ventricular under-sensing. These electrical characteristics translate into an increased burden of lead-related complications during follow-up, including threshold rise, intermittent or permanent loss of capture, and need for lead revision or system reprogramming.^4,5^

Across observational studies, long-term lead revision rates with HBP range between 7% and 11%, primarily driven by electrical instability and progressive threshold elevation.^77^ In addition, Vijayaraman *et al.* reported a 5-year generator replacement rate of approximately 9%, reflecting increased pacing outputs and accelerated battery depletion.^78^ Meta-analytical comparisons of CSP modalities suggest overall complication rates of around 5% with HBP, with lead failure and system revision representing the predominant contributors.^79^ Although acute procedural complications are uncommon, the limited long-term durability of HBP remains a relevant consideration, particularly in CRT candidates with advanced conduction disease or septal fibrosis.

LBBAP was developed to address several of the technical and electrical limitations associated with HBP by targeting a broader pacing region, distal to the His Bundle.^4^ Observational data consistently report higher implantation success rates, lower and more stable capture thresholds, and superior sensing performance with LBBAP.^43^ In pooled analyses, overall complication rates with LBBAP are approximately 2%, significantly lower than those reported with HBP.^50^ Lead-related complications, including lead failure or deactivation, appear less frequently, while reported lead dislodgement rates remain low, typically 1–2%, and comparable to those observed with HBP.^43,50^

In parallel, the adoption of CSP techniques is influenced by a measurable learning curve. Available data indicate that LBBAP is associated with a shorter and more reproducible learning curve compared to HBP, with procedural and fluoroscopy times progressively decreasing as operator experience increases.^10^ In a single-centre study, procedural performance appears to stabilize after approximately 45 cases.^80^ However, larger, multicentre data from MELOS study demonstrate that implantation success, electrical and fluoroscopic parameters continue to improve over the first 100 cases, before reaching a plateau.^43^

However, LBBAP introduces specific procedural risks related to deep septal lead implantation. Reported complications include septal perforation, septal branch injury, transient or persistent RBBB, and, rarely, acute or delayed LV perforation.^81^ Although the incidence of these events is low in experienced centres, concerns persist regarding the long-term implications of deep septal lead positioning, including thrombo-embolic risk, septal fibrosis, and the feasibility of lead extraction.^82^ Importantly, most LBBAP-related complications are reported during the acute or early post-implantation period, whereas late lead failure appears less frequent compared with HBP.^79^

Beyond deep septal implantation-related events, potential effects on tricuspid valve function should be considered, as the pacing lead crosses the valve and is positioned close to the annulus. Across observational studies, progression of tricuspid regurgitation (TR), following LBBAP, has been reported in 7.3–32.6% of patients, and it appears to be primarily related to mechanical interaction between the lead and the tricuspid valve apparatus, especially with a more basal lead position.^83–85^ At the same time, TR improvement has been described in a subset of patients, possibly reflecting improved ventricular function and restoration of atrioventricular conduction.^86^

Comparative safety data between CSP and BiV-CRT suggest that CSP, particularly LBBAP, is associated with similar or lower complication rates. In a propensity score-matched multicentre registry of CRT candidates, device-related complications occurred in approximately 4–5% in patients with LBBAP compared with 12–13% in those receiving BiV-CRT, with the excess risk in the BiV-CRT group largely driven by CS lead-related events, including lead dislodgment and phrenic nerve stimulation, as well as the higher infection risk associated with three-lead systems.^60^

Direct comparisons between LBBAP and HBP are derived primarily from pooled analyses of multicentre observational cohorts. These consistently demonstrate lower overall and lea-related complication rates with LBBAP, with reported complication rates of approximately 2% for LBBAP vs. 5% for HBP. While both techniques are associated with a low incidence of acute procedural complications, available follow-up data favour LBBAP as the more durable CSP strategy in CRT populations.^79^

To date, no RCT has been specifically designed with complications as primary endpoint for head-to-head comparison of CSP modalities. Consequently, conclusions regarding relative safety should be interpreted in the context of observational evidence, centre experience, and evolving implantation techniques.

## Controversies and future perspectives

Beyond these technical considerations, CSP also presents conceptual controversies. For instance, LVSP has been shown to improve interventricular synchrony more effectively than LBB pacing in bradycardia patients, but at the cost of delayed LV lateral wall activation; short-term haemodynamic and electromechanical benefits appear comparable to BVP and HBP.^20^ Since LBBAP requires additional electrophysiologic manoeuvres and surrogate criteria to confirm conduction system capture, it remains to be determined whether LVSP alone could achieve similar long-term clinical outcomes in CRT candidates.^20^

Additionally, patient substrate plays a key role in determining the feasibility and effectiveness of different pacing strategies. Extensive septal fibrosis, hypertrophy, or calcification as encountered in ischaemic heart disease or severe aortic stenosis may limit lead advancement and conduction system capture during LBBAP.^87^ Observational data have shown lower implantation success in those patients, highlighting that anatomical and histological characteristics can be a major procedural barrier. Importantly, these limitations may be apparent during the procedure, underscoring the need for procedural flexibility and for implanters to be proficient in both CSP and BVP techniques.

Currently, CSP, especially LBBAP, is conducted with pacing leads not originally engineered for this specific purpose.^88^ Advancements in dedicated tools and procedural techniques will be necessary to enhance implantation success rates and optimize long-term safety.

Innovative device integrations are also under exploration. In the CROSS-LEFT pilot study, LBBAP leads connected to DF-1 dual-chamber implanted cardioverter-defibrillators (ICDs) provided both reliable ventricular arrhythmia sensing and effective electromechanical resynchronization.^89^ In parallel, recent advances in transvenous ICD technology have introduced ultra-low-profile, catheter-delivered defibrillation leads, enabling more targeted ventricular placement. Incorporating defibrillation capabilities into LBBAP systems could, in the future, enable simultaneous CRT delivery and anti-tachycardia therapy using a single lead platform.^90^

*Table 3* provides a clinical perspective on the role of CSP in CRT, including current standards, evidence-based, hybrid strategies, special scenarios, and future directions.

**Table 3:** Clinical perspective on conduction system pacing (CSP) in cardiac resynchronization therapy (CRT)

ASPECT	KEY POINTS
**CURRENT STANDARD**	BVP is the guideline-recommended CRT for HF with LBBB, QRS ≥150 ms, LVEF <35% despite optimal therapy. Demonstrated survival benefit, HF hospitalization reduction, and reverse remodelling. Limitations: coronary sinus anatomy, non-physiological activation, procedural risks, ∼30% non-responders.
**CSP AS AN ALTERNATIVE**	HBP and LBBAP restore near-normal physiological ventricular activation via the His–Purkinje system. Early evidence shows equal or superior electro-mechanical resynchronization vs. BVP. LBBAP: lower capture thresholds, higher R-wave amplitudes, more stable parameters. HBP: most physiological but technically challenging with higher thresholds and sensing issues.
**EVIDENCE BASE**	Mostly observational data and small RCTs; large trials pending. Guidelines: CSP recommended (Class IIb) when BVP is not feasible or fails. Both LBBAP and HBP improve LVEF, shorten QRS, and enhance functional status. ESC/EHRA Consensus Statement 2025: Recognizes CSP as physiologic pacing; LBBAP preferred over HBP; CRT indication in LBBB; ‘reasonable’ option in non-LBBB, PICM, AF with AVNA
**HYBRID STRATEGIES**	HOT-CRT (HBP + LV pacing) and LOT-CRT (LBBAP + LV pacing) Greater QRS narrowing, better LVEF improvement, and fewer complications vs. BVP alone.
**LIMITATIONS AND CONTROVERSIES**	HBP: high thresholds, low R-wave amplitude, late threshold rise, ∼11% lead revision rate. LBBAP: long-term safety unknown; risks include septal perforation, myocardial injury, and extraction uncertainty. Limited data in ischaemic/infiltrative cardiomyopathy.
**FUTURE DIRECTIONS**	Development of dedicated CSP tools/leads to improved safety and success. Integration of defibrillation/anti-tachycardia therapy into CSP leads. Large RCTs (e.g. left vs. left trial) to define CSP’s first-line role. Likely future: complementary use of CSP and BVP tailored to patient conduction profiles.

*Abbreviations:* AF, atrial fibrillation; AV, atrioventricular; BVP, biventricular pacing; CRT, cardiac resynchronization therapy; CSP, conduction system pacing; HOT-CRT, His-optimized cardiac resynchronization therapy; HF, heart failure; HBP, His bundle pacing; ICD, implantable cardioverter-defibrillator; IVCD, intraventricular conduction delay; LBBB, left bundle branch block; LBBAP, left bundle branch area pacing; LOT-CRT, left bundle branch area pacing-optimized cardiac resynchronization therapy; LV, left ventricle/ventricular; LVEF, left ventricular ejection fraction; LVSP, left ventricular septal pacing; MRI, magnetic resonance imaging; NICM, non-ischaemic cardiomyopathy; NYHA, New York Heart Association; PICM, pacing-induced cardiomyopathy; RBBB, right bundle branch block; RCT, randomized controlled trial; RVP, right ventricular pacing.

## Limitations

This systematic review has several limitations that should be considered when interpreting the findings. Evidence for CSP-CRT is largely observational with small pilot RCTs, variable inclusion criteria, and limited follow-up, so effect estimates are imprecise and vulnerable to confounding and selection bias. Marked clinical and methodological heterogeneity existed, including mixed populations, diverse baseline conduction patterns, and variation in implantation targets, criteria for confirming His or left bundle capture, and use of hybrid HOT/LOT strategies. Capture success definitions were inconsistent, introducing misclassification and between-study variability.

Safety and lead-performance reporting was uneven and often short-term, limiting inference about durability, thresholds, extraction feasibility, and late complications. Publication and selective-reporting bias are possible because positive single-centre series from high-volume operators predominate, and learning-curve effects may reduce generalizability. In addition, potential overlap of patient cohorts across registry-based studies and subsequent subgroup analysis cannot be excluded, which may amplify observed effects. Comparative outcomes may also be influenced by operator and centre experience, as several studies report unusually low implantation success rates and long procedural times in the BVP arm, raising concerns for an imbalance in operator familiarity between pacing modalities.

Although a meta-analysis was not appropriate given the heterogeneity, reliance on structured narrative synthesis means conclusions were based on direction and consistency of effects rather than precise pooled effect sizes, underscoring the need for larger multicentre RCTs with standardized capture definitions and clinically focused endpoints.

## Conclusions

BVP remains the gold standard for CRT, yet CSP, including HBP and LBBAP, emerges as a physiologically appealing alternative capable of restoring near-normal ventricular activation in selected patients. However, current evidence is based on observational and small RCTs, with most reported benefits related to surrogate endpoints, rather than hard clinical outcomes. Therefore, larger, multicentre, prospective RCTs with extended follow-up, such as left vs. left and HOPE-HF II, are required to define its clinical role.

Individualized patient selection remains essential, based on conduction abnormality, ventricular activation pattern, and anatomical feasibility. Operator and centre experience could also influence procedural success and clinical outcomes, highlighting the need for adequate expertise in CSP techniques. CSP may be advantageous in unsuccessful CS lead implantation, in BiV-CRT non-responders, or in non-LBBB conduction disturbances. (Central Illustration) Ongoing development in dedicated lead designs, hybrid approaches (HOT-CRT, LOT-CRT), and integration of defibrillation therapies may further enhance its clinical utility. Ultimately, as our understanding of electromechanical activation patterns deepens and as higher-quality evidence accumulates, CSP may assume an increasingly important role as a complementary strategy within contemporary CRT, rather than a universal replacement for BVP.

## Supplementary Material

euag122_Supplementary_Data

## Data Availability

The data underlying this article are available in the article and in its online Supplementary material.
