# Supplementary material for: Conduction system pacing for cardiac resynchronization therapy: a systematic review
Source: Europace. 2026 May 19;28(6):euag122. doi: 10.1093/europace/euag122 (PMC13255998; doi:10.1093/europace/euag122)
Supplement: euag122_Supplementary_Data [file euag122_supplementary_data.docx]

**Supplementary material**

1. **Objective**

PICO:

- **Population (P):**

Adults (≥18 years) with heart failure and guideline indications for CRT, including de novo CRT candidates and CRT non-responders/failed BiV-CRT upgrade.

- **Intervention (I):**

Conduction system pacing (CSP) used for CRT, specifically:

-His bundle pacing CRT (HBP-CRT)

-His-optimized CRT (HOT-CRT = HBP + LV lead)

-Left bundle branch area pacing CRT (LBBAP-CRT / LBBP-CRT)

-Left bundle branch optimized CRT (LOT-CRT = LBBAP + LV lead)

- **Comparator (C):**

-Conventional biventricular CRT (BiV-CRT)

- **Outcomes (O):**

-Primary: Electrical Resynchronization

-Secondary: Clinical Response, HF Hospitalizations, All-Cause Mortality, Procedural Success And Complications.

1. **Search algorithms (up to 15 November 2025)**

**PubMed (MEDLINE):**

(("Cardiac Resynchronization Therapy"[Mesh] OR "cardiac resynchronization therapy"[tiab] OR CRT[tiab]

OR "biventricular pacing"[tiab] OR "bi-ventricular pacing"[tiab] OR "resynchronization therapy"[tiab]))

AND (("conduction system pacing"[tiab] OR "physiologic pacing"[tiab] OR "physiological pacing"[tiab])

OR ("His bundle pacing"[tiab] OR "His-bundle pacing"[tiab] OR HBP[tiab]

OR "His optimized CRT"[tiab] OR "His-optimized CRT"[tiab] OR HOT-CRT[tiab] OR "HOT CRT"[tiab])

OR ("left bundle branch area pacing"[tiab] OR "left bundle branch pacing"[tiab]

OR LBBAP[tiab] OR LBBP[tiab]

OR "left bundle branch optimized CRT"[tiab] OR LOT-CRT[tiab] OR "LOT CRT"[tiab]

OR "left bundle pacing"[tiab])) AND ("Heart Failure"[Mesh] OR "heart failure"[tiab] OR HF[tiab] OR "cardiomyopathy"[tiab] OR "left ventricular dysfunction"[tiab] OR LVEF[tiab])

NOT (animals[mh] NOT humans[mh])

**Scopus:**

TITLE-ABS-KEY

("cardiac resynchronization therapy" OR "resynchronization therapy" OR CRT

OR "biventricular pacing" OR "bi-ventricular pacing")

AND

TITLE-ABS-KEY

("conduction system pacing" OR "physiologic pacing" OR "physiological pacing"

OR "His bundle pacing" OR "His-bundle pacing" OR HBP

OR "His optimized CRT" OR "His-optimized CRT" OR HOT-CRT OR "HOT CRT"

OR "left bundle branch area pacing" OR "left bundle branch pacing"

OR LBBAP OR LBBP OR "left bundle pacing"

OR "left bundle branch optimized CRT" OR LOT-CRT OR "LOT CRT")

AND

TITLE-ABS-KEY ("heart failure" OR HF OR cardiomyopathy OR "left ventricular dysfunction" OR LVEF OR "reduced ejection fraction")

**Cochrane Library (CENTRAL):**

#1 ("cardiac resynchronization" OR "resynchronization therapy" OR CRT OR "biventricular pacing" OR "bi-ventricular pacing")

#2 ("conduction system pacing" OR "physiologic pacing" OR "physiological pacing")

#3 ("His bundle pacing" OR "His-bundle pacing" OR HBP OR "His bundle pacemaker" OR "His-optimized CRT" OR "HOT-CRT" OR "HOT CRT")

#4 ("left bundle branch area pacing" OR "left bundle branch pacing" OR LBBAP OR LBBP OR "left bundle branch block pacing" OR "left bundle pacing"

OR "left bundle branch optimized CRT" OR "LOT-CRT" OR "LOT CRT")

#5 (#2 OR #3 OR #4)

#6 ("heart failure" OR HF OR "cardiomyopathy" OR "left ventricular dysfunction" OR LVEF OR "reduced ejection fraction")

#7 (#1 AND #5 AND #6)
